# COVID-19 infection prevention and control preparedness among healthcare workers in public hospitals in Yemen: a comprehensive assessment of knowledge, practices, perceptions, and institutional readiness

**DOI:** 10.3389/fpubh.2026.1949595

**Published:** 2026-10-29

**Authors:** Ola M. Nader Jarada, Abdulsalam M. Al-Mekhlafi, Mohammed Jafer, Mosa A. Shubayr, Lana Sayal, Fatimah Ali Hadadi, Alhussain Mohammad Hendi, Fatmah Ali Hazazi, Shadi Abdullah Hamzi, Mohammed M. Al Moaleem, Ibtisam Moafa

**Affiliations:** 1 Department of Epidemiology, Faculty of Medicine and Health Science, Al-Razi University, Sana’a, Yemen; 2 Department of Parasitology, Faculty of Medicine and Health Sciences, Sana’a University, Sana’a, Yemen; 3 Mohammed Jafer, Preventive Dental Science Department, College of Dentistry, Jazan University, Jazan, Saudi Arabia; 4 Mosa A. Shubayr. Preventive Dental Science Department, College of Dentistry, Jazan University, Jazan, Saudi Arabia; 5 Oral Medicine Department, Faculty of Dentistry, Al-Wataniya Private University, University of Aden, Syria, Yemen; 6 Department of Pharmacy, Jazan University Hospital, Jazan University, Jazan, Saudi Arabia; 7 Department of Nursing, Jazan University Hospital, Jazan University, Jazan, Saudi Arabia; 8 University Dental Hospital, College of Dentistry, Jazan University, Jazan, Saudi Arabia; 9 Department of Prosthetic Dental Science, College of Dentistry, Jazan University, Jazan, Saudi Arabia; 10 Preventive Dental Science Department, College of Dentistry, Jazan University, Jazan, Saudi Arabia

**Keywords:** COVID-19, healthcare workers, infection prevention and control, occupational health, Yemen

## Abstract

**Background and aim:**

Healthcare workers (HCWs) remain at increased risk of occupational exposure to COVID-19, making effective infection prevention and control (IPC) essential for protecting healthcare personnel and patients. Evidence about HCWs’ knowledge, practices, perceptions, and institutional preparedness remains limited in Yemen. This study aimed to assess HCWs’ knowledge, practices, perceptions, and organizational preparedness regarding COVID-19 IPC measures in public hospitals in Sana’a City, Yemen, and examine demographic and professional factors associated with these outcomes.

**Methods:**

A descriptive cross-sectional study was conducted among 386 HCWs selected using stratified random sampling from five public hospitals in Sana’a from September to November 2023. Data were collected using a validated structured questionnaire assessing knowledge, practices, perceptions, and workplace factors, complemented by an observational checklist evaluating institutional IPC preparedness. Descriptive statistics and chi-square tests were used to analyze the data. A *p*-value of < 0.05 was considered statistically significant.

**Results:**

Overall, 39.7% of participants demonstrated good knowledge, with substantial deficiencies observed in knowledge of SARS-CoV-2 transmission (7.5%), despite relatively better knowledge of disease concepts (61.9%) and treatment (54.5%). Good preventive practice was observed in 55.4% of participants, although compliance with personal protective equipment varied considerably. Only 8.0% demonstrated an overall positive perception toward COVID-19 prevention. Knowledge was significantly associated with age, educational level, profession, and years of experience (all *p* < 0.05), whereas practice was associated with sex, age, and profession. Observational findings revealed important institutional deficiencies, including the absence of negative-pressure isolation rooms, alcohol-based hand hygiene stations, routine HCW screening, and COVID-19-specific IPC guidelines.

**Conclusion:**

HCWs in Yemeni public hospitals demonstrated moderate preventive practices but insufficient knowledge, predominantly negative perceptions, and substantial institutional preparedness gaps. Strengthening competency-based IPC training, ensuring continuous access to updated guidelines and essential resources, and improving organizational preparedness are critical for enhancing HCW safety and strengthening resilience against future infectious disease outbreaks.

## Introduction

Coronavirus disease 2019 (COVID-19), caused by severe acute respiratory syndrome coronavirus 2 (SARS-CoV-2), emerged in late 2019 and rapidly evolved into a global public health emergency (1, 2). Although the World Health Organization (WHO) declared the end of COVID-19 as a Public Health Emergency of International Concern in May 2023, SARS-CoV-2 continues to circulate worldwide, with periodic outbreaks and the emergence of new variants posing ongoing challenges to healthcare systems and infection prevention programs (3).

COVID-19 is primarily transmitted through respiratory droplets and aerosols generated during coughing, sneezing, speaking, or breathing, particularly in crowded or poorly ventilated indoor environments (4, 5). The incubation period typically ranges from 2 days to 14 days (6), and clinical manifestations vary from asymptomatic infection to fatigue, exertional dyspnea, concentration or memory disturbance, and sleep problems (7).

Healthcare workers (HCWs) have remained among the occupational groups at greatest risk of COVID-19 because of their continuous exposure to infected patients and contaminated clinical environments (8). Beyond increasing the likelihood of infection, occupational exposure has contributed to healthcare-associated transmission, workforce shortages, psychological distress, burnout, and disruption of essential healthcare services (9, 10). Consequently, protecting HCWs has remained a cornerstone of pandemic preparedness and health system resilience (11).

The implementation of effective infection prevention and control (IPC) measures is essential for minimizing occupational transmission of SARS-CoV-2 within healthcare facilities. International guidelines recommend a comprehensive IPC strategy that includes appropriate use of personal protective equipment (PPE), strict hand hygiene, respiratory etiquette, environmental cleaning and disinfection, adequate ventilation, patient triage, vaccination, and continuous education and competency-based training of healthcare personnel (3, 12, 13).

Evidence from recent systematic reviews indicates that constant adherence to IPC recommendations is connected with reduced healthcare-associated transmission, enhanced HCW care, and supported health system awareness for infectious disease outbreaks (14, 15).

Despite the availability of evidence-based IPC guidelines, adherence among HCWs remains suboptimal and inconsistent. This gap is particularly evident in low- and middle-income countries, where structural and systemic constraints, such as shortages of PPE, inadequate IPC training, limited financial and material resources, and heavy clinical workloads, continue to compromise the effective implementation of IPC practices. Recent systematic reviews have consistently highlighted these barriers as key determinants of poor compliance in resource-limited healthcare settings. Furthermore, HCWs’ knowledge, attitudes, perceptions, and workplace environment have been identified as critical factors influencing adherence to IPC recommendations, playing a central role in reducing occupational exposure to infectious diseases and improving patient and provider safety (16, 17).

These challenges are particularly evident in fragile and conflict-affected settings such as Yemen. Years of armed conflict have severely weakened the country’s healthcare infrastructure, resulting in shortages of healthcare personnel, essential medical supplies, diagnostic capacity, and infection prevention resources. These longstanding systemic deficiencies have complicated the implementation of IPC measures during the COVID-19 pandemic and increased occupational risks among HCWs (18, 19).

Although few studies have investigated COVID-19-related knowledge, attitudes, and practices among Yemeni HCWs, most were web-based cross-sectional surveys conducted during the early phases of the pandemic. These studies largely relied on self-reported data and primarily assessed perceived knowledge and attitudes, with limited evaluation of actual clinical practices and workplace environmental conditions, thereby restricting a comprehensive understanding of IPC implementation in real-world healthcare settings (20, 21).

Moreover, evidence derived from direct observation within public hospitals remains scarce because most studies in low- and middle-income settings rely on self-reported data, thus creating a knowledge gap regarding real infection prevention practices in daily clinical settings (17).

Therefore, this study aimed to comprehensively assess occupational health and IPC practices among HCWs in public hospitals in Sana’a City, Yemen. Specifically, the study evaluated: (1) the availability and appropriate utilization of PPE; (2) HCWs’ knowledge, practices, and perceptions regarding COVID-19 infection prevention; and (3) environmental and occupational risk factors associated with infection control practices. Furthermore, the study examined whether demographic and professional characteristics, including age, sex, educational level, occupation, and years of professional experience, were associated with variations in knowledge, practices, and perceptions related to COVID-19. The null hypothesis was that no significant associations exist between these demographic or professional characteristics and HCWs’ knowledge, practices, or perceptions regarding COVID-19 IPC measures.

## Materials and methods

### Study design and setting

A descriptive cross-sectional study was conducted to assess occupational health and infection prevention and control (IPC) practices related to COVID-19 among healthcare workers (HCWs) in public hospitals in Sana’a City, Yemen. The study focused on knowledge, practices, perceptions, and environmental determinants of IPC compliance. Data were collected from five public hospitals from September 2023 to November 2023.

### Study setting, population, and eligibility criteria

The study population included all licensed male and female HCWs employed in five major public hospitals in Sana’a City. The selected facilities represented major tertiary and secondary healthcare facilities and served a large proportion of the population. Participants who did not meet the inclusion criteria or whose questionnaires contained substantial incomplete responses were excluded.

### Ethical considerations

Ethical approval was obtained from the Faculty of Medical Sciences at Al Razi University, Yemen (Reference No. RU/16/FMHS, 4/8/2020), prior to commencement of the study. Official permissions were secured from the administrations of the participating hospitals before data collection. Participants provided written informed consent after the study objectives and procedures were explained. Participation was voluntary, and participants were informed that they could withdraw from the study at any stage without consequences. Confidentiality and anonymity were maintained by removing personal identifiers. Data were stored securely in password-protected systems and were accessible only to the research team.

### Sample size and sampling technique

An *a priori* power analysis was performed to determine the minimum sample size required for the planned analyses of associations between healthcare workers’ (HCWs’) demographic and professional characteristics and the main study outcomes, including knowledge, practices, and perceptions regarding infection prevention and control (IPC). The calculation was based on a chi-square test of independence, assuming a small-to-moderate effect size (Cohen’s *w* = 0.20), a two-sided significance level of 0.05, 80% statistical power, and 3 degrees of freedom. Under these assumptions, the minimum required sample size was estimated at approximately 273 participants. The target sample was increased to 386 HCWs to provide adequate representation across the participating hospitals and professional categories and to account for potential nonresponse and incomplete questionnaires.

The total population of eligible HCWs across the five selected public hospitals was 5,933 and served as the sampling frame for proportional allocation. A stratified random sampling technique was used, with hospital and professional category serving as stratification variables. Hospitals constituted the primary sampling strata, while HCWs were further stratified according to professional category. The required sample of 386 HCWs was allocated proportionally according to the number of eligible HCWs in each hospital–professional stratum.

Within each stratum, eligible HCWs were selected using a random sampling procedure from the available staff lists provided by the participating hospitals. This approach was used to minimize over- or under-representation of particular hospitals or professional groups and to ensure that the sample broadly reflected the underlying distribution of eligible HCWs across the participating hospitals and professional categories.

The final numbers of HCWs selected from the five hospitals were 189, 50, 19, 86, and 42, respectively, giving a total sample of 386 participants. No sampling or post-stratification weights were applied during the statistical analysis because proportional allocation was incorporated into the sampling design. Consequently, the reported estimates represent the observed study sample and were not subsequently weighted to reproduce the underlying distribution of HCWs across hospitals or professional categories.

Potential nonresponse bias was considered because participation was voluntary and some eligible HCWs may have declined participation or may not have been available during the data-collection period. Questionnaires were distributed during both morning and evening shifts to maximize coverage of HCWs working different schedules and to reduce potential nonresponse related to shift patterns. However, individual-level information on nonrespondents was not available; therefore, a formal statistical comparison between respondents and nonrespondents could not be performed. Consequently, residual nonresponse bias cannot be completely excluded. Participants who responded may have differed from those who declined or were unavailable, particularly with respect to workload, IPC training, professional responsibilities, knowledge, or practices. The 39 participants involved in the pilot study were separate from the main study sample and were not included among the 386 HCWs included in the main study or in the final statistical analysis.

### Study instrument and questionnaire development

The study questionnaire was developed on the basis of an extensive review of previously published literature and relevant WHO guidance documents, including the WHO clinical management guidelines for COVID-19 and occupational risk assessment frameworks for HCWs. In addition, more recent WHO updates on IPC practices and HCW safety during respiratory infectious disease outbreaks were considered to ensure alignment with current evidence-based standards (22, 23).

The questionnaire was initially developed in English and subsequently translated into Arabic using a standardized forward–backward translation process conducted by two independent bilingual translators to ensure conceptual equivalence, linguistic accuracy, and cultural relevance. The pre-final Arabic version was reviewed by the research team.

A pilot study was conducted among 39 participants, corresponding to approximately 10% of the planned sample size. These participants were not included in the final analysis. The pilot study was conducted to assess the clarity, relevance, comprehensibility, and acceptability of the questionnaire items and to identify any ambiguous or difficult-to-understand questions. Based on the participants’ feedback, minor modifications were made to improve the wording and clarity of the questionnaire before the final version was administered in the main study.

The initial item pool was compiled and subsequently reviewed by a multidisciplinary expert panel to assess content validity, clarity, and relevance to the study objectives. The final instrument consisted of closed-ended questions covering key domains of knowledge, perceptions, practices, and environmental factors related to COVID-19.

#### Observational checklist

In addition to the self-administered questionnaire, an observational checklist was used to assess selected infection prevention and control (IPC) measures in each participating hospital. The checklist was developed based on relevant World Health Organization (WHO) recommendations and previously published IPC assessment instruments addressing COVID-19-related infection prevention and occupational safety measures (22, 23). It primarily assessed observable institutional, environmental, and organizational IPC measures, including the availability of personal protective equipment (PPE), hand hygiene facilities, environmental and workplace safety measures, triage arrangements, masking provisions, and other institutional IPC resources. The checklist was intended to complement the questionnaire-based assessment and was not designed to systematically measure individual healthcare workers’ adherence to specific IPC behaviors.

The checklist items were reviewed by the multidisciplinary expert panel involved in the development of the study instruments to assess their relevance, clarity, and alignment with the study objectives. Before the main data collection, the checklist was pretested to identify unclear or difficult-to-observe items, and minor modifications were made to improve clarity and consistency. Before data collection, the observers received standardized instructions regarding the interpretation and application of each checklist item. The same assessment procedures and criteria were followed across all participating hospitals to promote consistency in data collection.

A formal inter-rater reliability assessment was not performed during the original data collection; therefore, no inter-rater reliability coefficient was calculated or reported. The WHO Infection Prevention and Control Assessment Framework (IPCAF) was not used as the primary observational instrument, as data collection was conducted using the predefined study-specific checklist. The use of a study-specific checklist and the absence of formal inter-rater reliability assessment are acknowledged as methodological limitations of the observational component of the study.

#### Questionnaire parts

The questionnaire was divided into six sections:

Part I: Sociodemographic and occupational characteristics: This section included sociodemographic and occupational characteristics, including sex, age, educational level, years of professional experience, professional category, and specialty. For the primary analyses, age was categorized into two groups (20–25 years and >25 years), while years of professional experience were categorized as 1–3 years and >3 years. These predefined categories were applied consistently in the subsequent analyses.

Part II: IPC training and observational assessment: This section assessed participation in IPC training programs and institutional IPC implementation using four yes/no questions. In addition, an observational checklist was applied in each participating hospital to assess key observable IPC measures, as described above.

Part III: Knowledge of COVID-19: This section consisted of 46 scored items assessing knowledge of COVID-19, divided into five content domains: concept (seven items), transmission (seven items), signs and symptoms (17 items), prevention (eight items), and treatment (seven items). Responses were multiple-choice and categorized as “correct,” “incorrect,” or “I don’t know.”

Part IV: Practices related to COVID-19: This section comprised 14 scored practice items derived from four questions assessing preventive practices. Three questions included multiple response options and were further subdivided into several scored items. Responses were coded as correct, incorrect, or “I don’t know,” as appropriate.

Part V: Perceptions toward COVID-19: This section comprised 15 items assessed using a five-point Likert scale ranging from strongly disagree to strongly agree.

Part VI: Environmental and workplace factors: This section contained 10 yes/no items assessing environmental and workplace factors influencing IPC compliance within healthcare settings.

#### Data collection

An information sheet explaining the study’s purpose was provided to all participants prior to data collection. The questionnaire required approximately 15 min to complete. All responses were coded, anonymized, and securely stored to ensure confidentiality and facilitate statistical analysis.

The finalized Arabic, self-administered questionnaire was distributed directly to eligible HCWs during routine working hours across morning and evening shifts to maximize participant coverage. Completed questionnaires were collected on the same day and reviewed daily for completeness and consistency before data entry. Questionnaires with substantial missing data or incomplete responses were excluded in accordance with the predefined eligibility criteria.

#### Scoring system

Knowledge and practice scores were calculated as the percentage of correct responses. Each correct response was assigned a score of 1, whereas incorrect and “I don’t know” responses were assigned a score of 0. For knowledge, the overall score and the scores for the predefined knowledge domains were calculated based on the proportion of correct responses across the relevant items within each domain. Thus, classification of a participant as having good or poor knowledge within a specific domain was based on the aggregate score across all items comprising that domain, rather than on any individual item. A score of ≥70% was classified as good knowledge or practice, whereas a score of <70% was classified as poor knowledge or practice. This threshold was prespecified in the study scoring protocol and was applied consistently to all participants.

Perceptions toward COVID-19 IPC measures were assessed using 15 statements rated on a five-point Likert scale ranging from strongly disagree (1) to strongly agree (5), yielding a maximum possible score of 75. A total score of ≥51 was classified as a positive perception, whereas a score of ≤50 was classified as a negative perception. This cut-point was prespecified in the study scoring protocol and applied consistently across participants. For descriptive analyses of individual items, responses of strongly agree and agree were grouped as positive responses, whereas neutral, disagree, and strongly disagree responses were grouped as negative responses.

#### Data processing and statistical analysis

The answered questionnaires were screened for completeness before data entry. Questionnaires with substantial missing data were excluded in accordance with the predefined eligibility criteria. The remaining questionnaires were coded and entered into SPSS (version 20.0, IBM Corp., Armonk, NY, USA) for statistical analysis.

Descriptive statistics were used to summarize participants’ sociodemographic and occupational characteristics and their responses to the study questionnaire. Categorical variables were presented as frequencies and percentages, whereas continuous variables were summarized as means and standard deviations (SDs). Graphical presentations, including bar charts, were used to illustrate selected environmental and organizational factors.

The internal consistency of the questionnaire was assessed among the main study sample (*n* = 386) using Cronbach’s alpha coefficient. The overall questionnaire demonstrated good internal consistency, with a Cronbach’s alpha of 0.81. The knowledge items were organized into five content domains (concept, transmission, signs and symptoms, prevention, and treatment) to describe different areas of COVID-19 knowledge rather than independently validated psychometric scales. The perception section consisted of 15 Likert-scale items.

Multivariable logistic regression analyses were performed to identify factors independently associated with good knowledge, good infection-prevention practice, and positive perception. Hospital, sex, age group, occupation, educational level, and work experience were included as explanatory variables, with hospital incorporated as a fixed effect. Adjusted odds ratios (aORs) with 95% confidence intervals (CIs) were reported, and statistical significance was set at *p* < 0.05.

#### Sensitivity analysis of dichotomization thresholds

To assess the robustness of the prespecified dichotomization thresholds, sensitivity analyses were performed using alternative cut-points of 60 and 75% of the maximum possible score. The original classification thresholds were retained as the primary analysis: ≥33/46 for knowledge, ≥10/14 for practice, and ≥51/75 for perception. For the sensitivity analyses, scores corresponding to 60 and 75% of the maximum possible score were used as alternative thresholds. The resulting proportions of participants classified as having good knowledge, good infection-prevention practice, or positive perception were recalculated and compared across the alternative thresholds.

ROC-based threshold derivation and diagnostic sensitivity/specificity analyses were not performed because no independent external criterion or gold-standard outcome was available in the dataset.

#### Internal consistency and item analysis

In response to the reviewer’s comment, additional *post hoc* internal consistency analyses were performed using the item-level data from the 386 participants. Cronbach’s alpha coefficients were calculated separately for each predefined knowledge domain (concept, transmission, signs and symptoms, prevention, and treatment) and for the 15-item perception scale. For the knowledge domains, items were coded as 1 for a correct response and 0 for an incorrect or “I don’t know” response. For the perception scale, the original five-point Likert responses were retained for reliability analysis. Corrected item–total correlations and Cronbach’s alpha if item deleted were additionally calculated for the transmission knowledge domain to identify items contributing to the observed deficits. These analyses were exploratory and were performed to further characterize the internal consistency and item-level performance of the study instrument.

## Results

### Demographic characteristics

Figure 1 presents the demographic and professional characteristics of the participating HCWs. Of the 386 participants, 53.9% were female, and 58.5% were older than 25 years. Educational level and years of professional experience were broadly comparable across participants. Regarding professional category, laboratory personnel constituted the largest group (90, 23.3%), followed by nurses (84, 21.8%), whereas midwives represented the smallest group (12, 3.1%).

**Figure 1:**
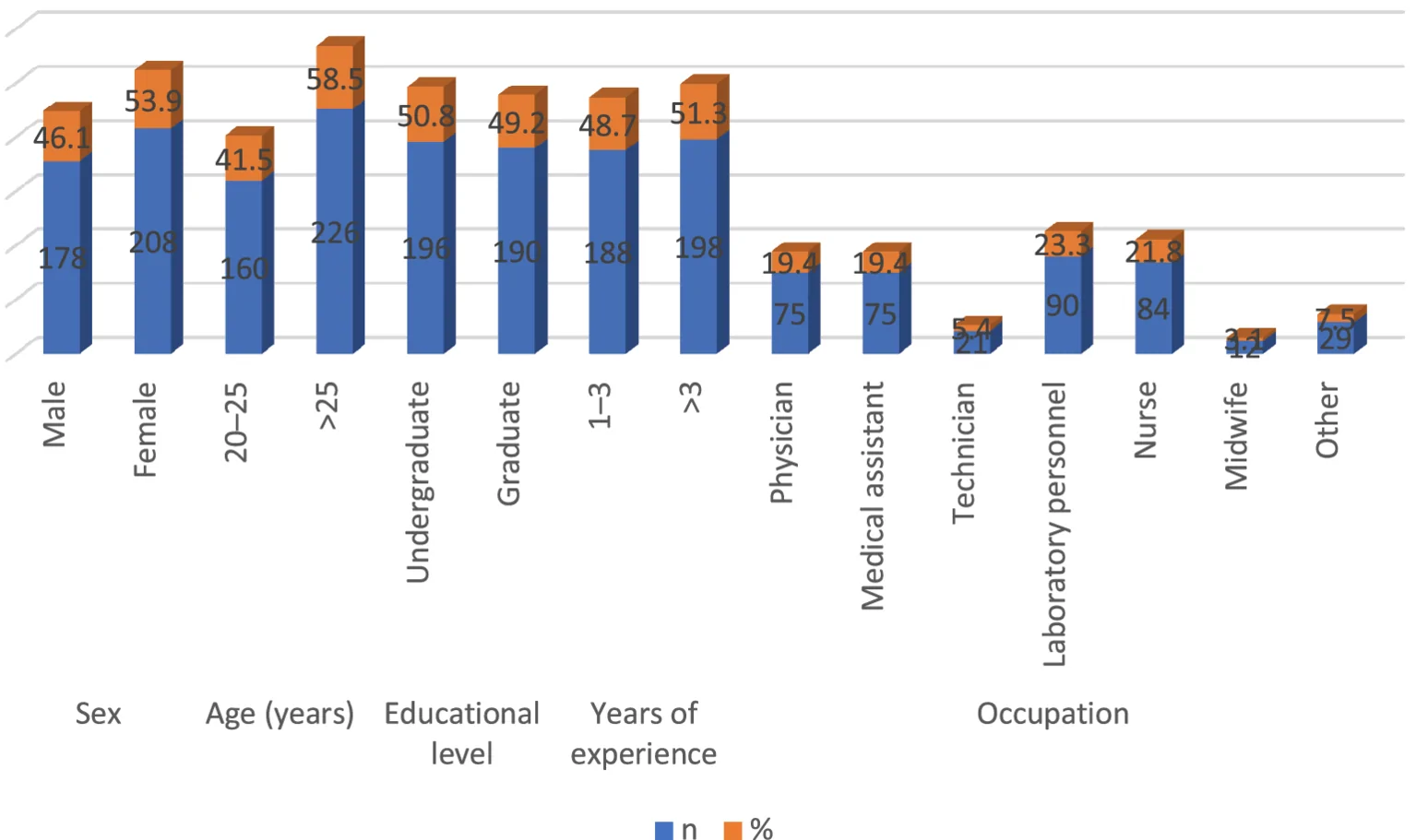
Sociodemographic characteristics of healthcare workers (*n* = 386).

### Questionnaire reliability

The study assessed the internal consistency of the questionnaire using Cronbach’s alpha. The overall questionnaire demonstrated good internal consistency, with an overall Cronbach’s alpha of 0.81. In the *post hoc* domain-specific analysis, Cronbach’s alpha coefficients were 0.426 for the concept domain, 0.626 for transmission, 0.746 for signs and symptoms, 0.245 for prevention, and 0.369 for treatment. The 15-item perception scale demonstrated a Cronbach’s alpha of 0.626 (Table 1).

**Table 1:** Internal consistency of the knowledge domains and perception scale assessed using Cronbach’s alpha.

Domain	No. of items	Cronbach’s *α*
Concept	7	0.426
Transmission	7	0.626
Signs and symptoms	17	0.746
Prevention	8	0.245
Treatment	7	0.369
Perception	15	0.626

The knowledge domains were defined primarily as content domains covering different aspects of COVID-19 knowledge rather than as independently validated psychometric subscales. Therefore, the domain-specific alpha coefficients, particularly those for prevention and treatment, were interpreted cautiously.

Because transmission knowledge represented the most prominent area of deficit, an exploratory item-level analysis was performed for the seven transmission items (Table 2). Correct response rates varied substantially across items. Respiratory droplets were correctly identified by 96.4% of participants, whereas correct responses were considerably lower for airborne transmission (28.2%), wild animals (27.7%), contaminated surfaces or tools (5.7%), and undercooked food or meat (37.3%). Correct responses for contaminated water and blood transfusion were 56.5 and 50.0%, respectively.

**Table 2:** Item-level analysis of the COVID-19 transmission knowledge domain.

Transmission item	Correct (%)	Corrected item–total correlation	*α* if item deleted
Respiratory droplets	96.4	0.032	0.648
Airborne transmission	28.2	0.249	0.620
Contaminated water	56.5	0.529	0.513
Blood transfusion	50.0	0.515	0.519
Wild animals	27.7	0.361	0.581
Contaminated surfaces/tools	5.7	0.134	0.636
Undercooked food/meat	37.3	0.436	0.552

Corrected item–total correlations ranged from 0.032 to 0.529. The respiratory-droplet item had the lowest corrected item–total correlation (0.032), reflecting the very high proportion of participants who answered this item correctly. Removing this item would increase Cronbach’s alpha for the transmission domain only modestly, from 0.626 to 0.648. Other items showed corrected item–total correlations ranging from 0.134 to 0.529, with alpha-if-item-deleted values ranging from 0.513 to 0.636.

These findings indicate substantial heterogeneity in transmission-related knowledge. Importantly, the low overall performance of the transmission domain was driven by poor recognition of several specific transmission-related items rather than by the respiratory-droplet item alone. Domain classification was based on the aggregate score across all transmission items rather than on individual item responses.

### Organizational factors related to COVID-19 IPC

Figure 2 summarizes the organizational factors related to COVID-19 IPC across the participating hospitals on the basis of the observational checklist. Two of the five participating hospitals had established IPC programs and provided regular IPC training for HCWs. By contrast, none of the participating hospitals had implemented a routine COVID-19 screening strategy for HCWs or had COVID-19-specific IPC guidelines at the time of data collection.

**Figure 2:**
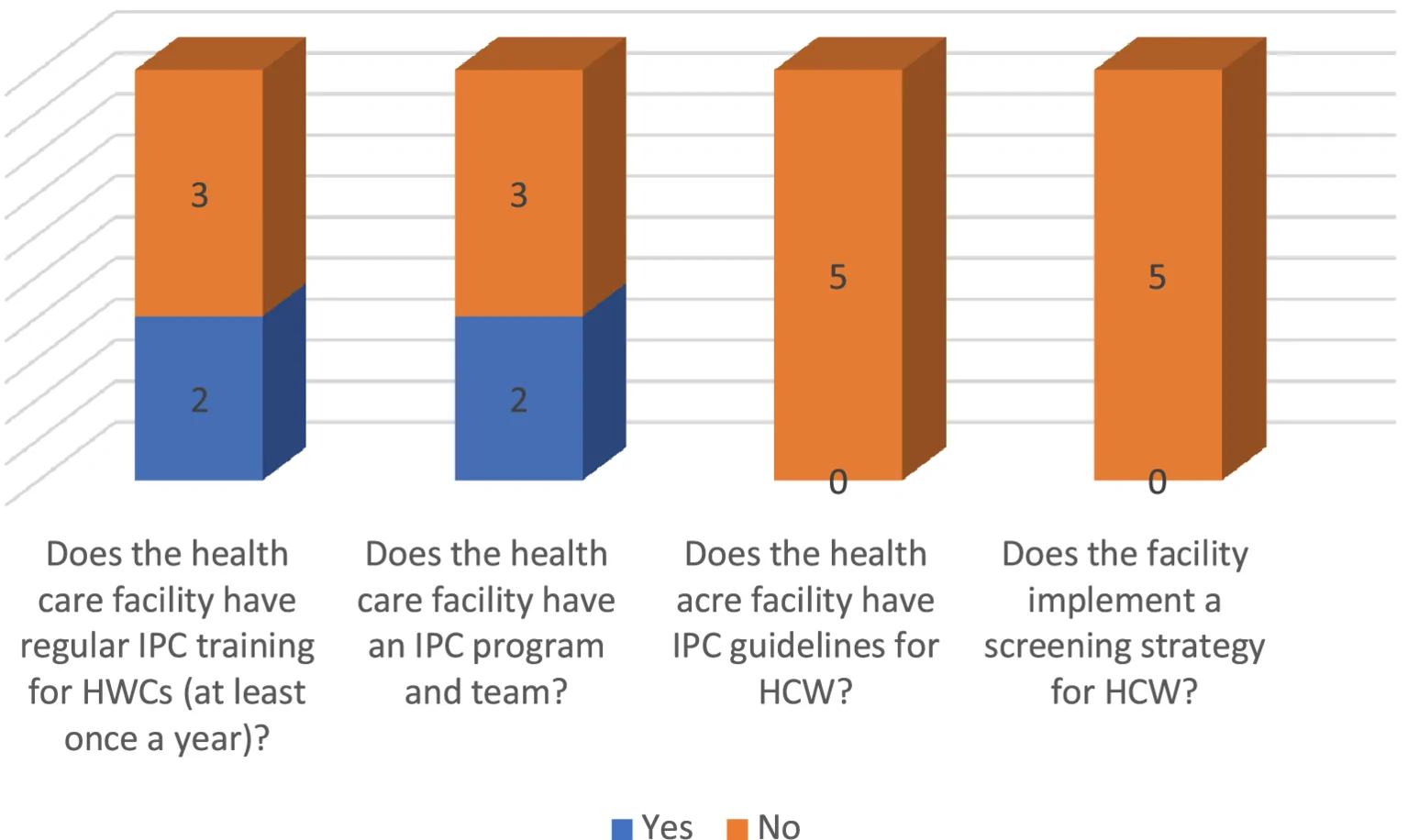
Organizational factors related to COVID-19.

### Knowledge of HCWs regarding COVID-19

Figure 3 illustrates HCWs’ knowledge of COVID-19. Among the assessed knowledge domains, the highest proportion of participants demonstrating good knowledge was observed for the concept of COVID-19 (61.9%), followed by treatment (54.5%). Knowledge of COVID-19 transmission modes was the poorest-performing domain, with only 7.5% of HCWs demonstrating good knowledge. Regarding overall knowledge, 39.4% (152/386) of participants had good knowledge, whereas 60.6% (234/386) had poor knowledge. Overall, these findings indicate variability in knowledge across different domains, with relatively stronger understanding of conceptual and treatment aspects and notable gaps in transmission-related knowledge.

**Figure 3:**
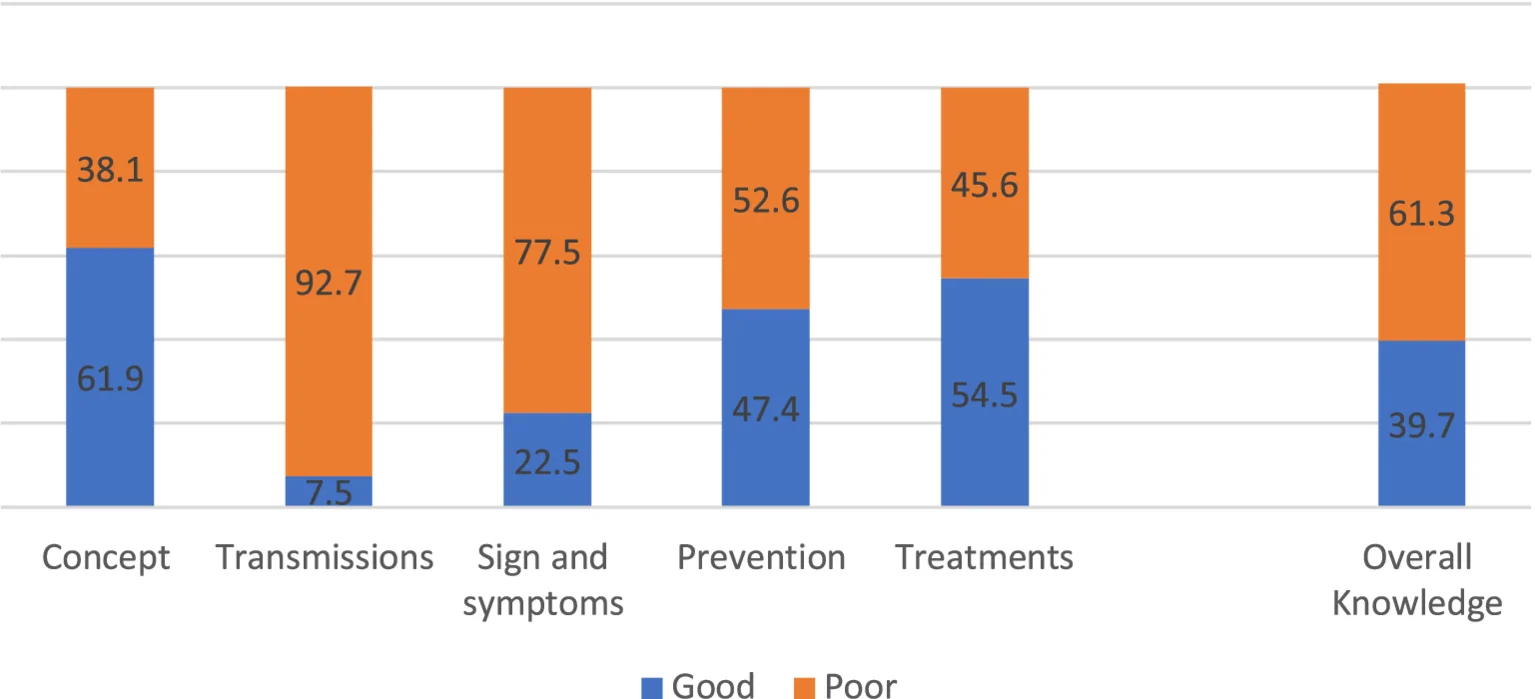
Distribution of knowledge levels across assessed domains among participants.

Regarding the concept of COVID-19 disease, most HCWs provided correct answers to five questions, with correct response rates ranging from 95.10 to 86.8%. Only two questions had lower correct response rates at 62.70 and 56.50%. The highest percentages of incorrect answers were observed for “COVID-19 is an infectious disease caused by an emerging type of the corona virus family” and “The incubation period of COVID-19 is 5–6 days and can be up to 14 days,” with incorrect response rates of 4.90 and 7.80%, respectively (Table 3).

**Table 3:** Distribution of healthcare workers’ knowledge regarding COVID-19 (*n* = 386).

Knowledge items	Correct	Incorrect/I do not know
	*n* (%)	*n* (%)
Concept		
Covid-19 is an infectious disease caused by an emerging type of the corona virus family.	367 (95.10)	19 (4.90)
COVID-19 is a disease similar to a respiratory system disease, such as SARS and MERS, in terms of symptoms and prevalence.	335 (86.80)	51 (13.20)
The incubation period of COVID-19 is 5–6 days and can be up to 14 days.	356 (92.20)	30 (7.80)
The clinical manifestations of COVID-19 are generally milder in children than in adults.	242 (62.70)	144 (37.30)
All persons with suspected, probable, or confirmed COVID-19 should be immediately isolated.	341 (88.30)	45 (11.70)
All suspect cases should be tested to determine if they are a confirmed case.	350 (90.70)	36 (9.30)
A mild case is a symptomatic patient meeting the case definition for COVID-19 without evidence of viral pneumonia or hypoxia.	218 (56.50)	168 (43.50)
Transmissions		
Respiratory droplets of infected individuals during coughing and sneezing	372 (96.4)	14 (3.6)
Air	109 (28.2)	277 (71.8)
Contaminated water	218 (56.5)	168 (43.5)
Blood transfusion from affected person	193 (50.0)	193 (50.0)
Contact with infected wild animals	107 (27.7)	279 (72.3)
Contact with contaminated surfaces and tools as a direct method of transmission	22 (5.7)	364 (94.3)
Eat foods, especially meat and eggs, without cooking them well	144 (37.3)	242 (62.7)
Signs and symptoms		
Sore throat	349 (90.4)	37 (9.6)
Cough	371 (96.1)	15 (3.9)
Shortness of breath	377 (97.7)	9 (2.3)
Chills	247 (64.0)	139 (36.0)
Vomiting	153 (39.6)	233 (60.4)
Nausea	169 (43.8)	217 (56.2)
Diarrhea	175 (45.3)	211 (54.7)
Headache	330 (85.5)	56 (14.5)
Rash	35 (9.1)	351 (90.9)
Conjunctivitis	138 (35.8)	248 (64.2)
Muscle ache	258 (66.8)	128 (33.2)
Joint ache	289 (74.9)	97 (25.1)
Loss of appetite	297 (76.9)	89 (23.1)
Loss of smell (anosmia) or taste	345 (89.4)	41 (10.6)
Nosebleed	54 (14.0)	332 (86.0)
Fatigue	225 (58.3)	161 (41.7)
Seizures	123 (31.9)	263 (68.1)
Prevention		
COVID-19 can be prevented by hand washing and personal hygiene.	368 (95.3)	18 (4.7)
For persons without symptoms, wearing a mask of any type is not recommended.	77 (19.9)	309 (80.1)
In the triage, a spatial distance of at least 1 m must be maintained, and no PPE is required.	224 (58.0)	162 (42.0)
In the patient room, HCWs must wear the following PPE		
Respirator N95 or FFP2	372 (96.4)	14 (3.6)
Gown	354 (91.7)	32 (8.3)
Gloves	378 (97.9)	8 (2.1)
Eye protection	355 (92.0)	31 (8.0)
Apron	340 (88.1)	46 (11.9)
Treatment		
Is WHO recommend screening all persons at first point of contact with the health system	360 (93.3)	26 (6.7)
Patients with mild and moderate illness may not require emergency interventions or hospitalization	254 (65.8)	132 (34.2)
WHO recommend for severe COVID-19 patients to use cautious fluid management	307 (79.5)	79 (20.5)
WHO recommends providing basic mental health and psychosocial support for all persons with suspected or confirmed COVID-19	358 (92.7)	28 (7.3)
WHO recommends patients with mild COVID-19 to		
Be given symptomatic treatment such as antipyretics for fever and pain	340 (88.1)	46 (11.9)
Have adequate nutrition	374 (96.9)	12 (3.1)
Have appropriate rehydration	291 (75.4)	95 (24.6)

As shown in Table 3, nearly all respondents (96.4%) correctly identified respiratory droplets as a transmission route. Others believed COVID-19 spreads through air, contaminated water, blood transfusion, contact with wild animals, contaminated surfaces and tools, and consuming undercooked food, particularly meat, with correct response rates of 28.2, 56.5, 50.0, 27.7, 5.7, and 37.3%, respectively.

For the signs and symptoms domain, the highest correct responses were observed for shortness of breath, cough, and loss of smell (anosmia), at approximately 90.0%, whereas the lowest correct response rates were observed for nosebleed (14.0%) and rash (9.1%). The highest percentages of incorrect responses were observed for rash and nosebleed (90.9 and 86.0%, respectively), whereas the lowest percentages of incorrect responses were observed for loss of smell (anosmia) or taste, sore throat, cough, and shortness of breath at 10.6, 9.6, 3.9, and 2.3%, respectively.

In relation to the prevention domain, 95.3% or more of participants provided correct responses regarding the prevention of COVID-19 through hand washing and personal hygiene. Correct response rates for the use of appropriate PPE in patient rooms ranged from 88.1 to 96.4%.

Table 3 presents the findings for the treatment domain of COVID-19. Correct responses were recorded for 93.3 and 92.7% of participants regarding “WHO recommends screening all persons at first point of contact with the healthcare system” and “WHO recommends providing basic mental health and psychosocial support for all persons with suspected or confirmed COVID-19,” respectively. For the statement “WHO recommends patients with mild COVID-19 to,” 88.1, 96.9, and 75.4% of participants correctly identified symptomatic treatment such as antipyretics for fever and pain, adequate nutrition, and appropriate rehydration, respectively.

Overall, knowledge was highest in the conceptual and treatment domains, whereas substantial gaps remained in transmission and symptom recognition.

### Practice of HCWs regarding COVID-19

Overall, 182 (47.2%) HCWs demonstrated good practice toward COVID-19, whereas 204 (52.8%) exhibited poor practice according to the prespecified 70% cut-off (Figure 4). As presented in Table 4, adherence to recommended hand hygiene practices was reported by 298 (77.2%) participants. Regarding WHO’s “Five Moments for Hand Hygiene,” the highest level of compliance was observed after touching a patient (85.8%), followed by before touching a patient (84.5%), after the risk of body fluid exposure (80.6%), and before clean/aseptic procedures (80.1%). Compliance with IPC standard precautions when in contact with any patient was reported by 252 (65.3%) HCWs.

**Figure 4:**
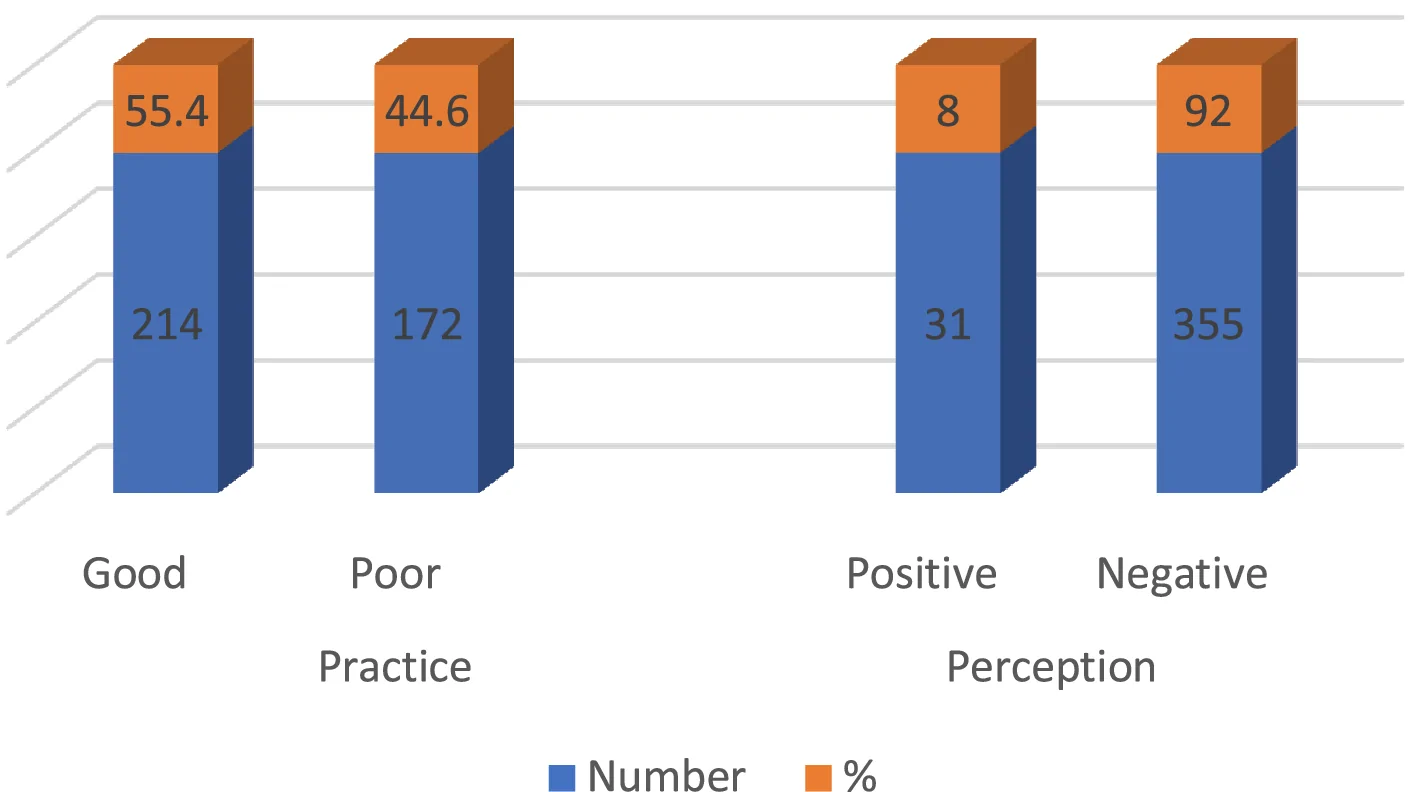
Overall practice and perceptions of participants in relation to COVID-19.

**Table 4:** Distribution of healthcare worker practice regarding COVID-19 (*n* = 386).

Practice items	Good practice	Poor practice
	*n* (%)	*n* (%)
Do you follow recommended hand hygiene practices?	298 (77.2)	88 (22.8)
Do you use alcohol-based hand rub or soap and water in the following cases?		
Before touching a patient	326 (84.5)	60 (15.5)
Before cleaning/aseptic procedures	309 (80.1)	77 (19.9)
After (risk of) body fluid exposure	311 (80.6)	75 (19.4)
After touching a patient	331 (85.8)	55 (14.2)
Do you follow IPC standard precautions when in contact with any patient?	252 (65.3)	134 (34.7)
Do you wear the following PPE when dealing with patients?		
Medical/surgical mask	274 (71.0)	112 (29.0)
Face shield	234 (60.6)	152 (39.4)
Gloves	305 (79.0)	81 (21.0)
Goggles/glasses	178 (46.1)	208 (53.9)
Gown	208 (53.9)	178 (46.1)
Coverall	197 (51.0)	189 (49.0)
Head cover	203 (52.6)	183 (47.4)
What is the WHO-recommended number of hand hygiene moments for healthcare workers?		
I do not know	142 (36.8%)	
Three times	120 (31.1%)	
Four times	35 (9.1%)	
Five times	41 (10.6%)	
Six times	48 (12.4)	

With respect to PPE, the highest proportion of participants reported routinely wearing gloves (79.0%), followed by medical/surgical masks (71.0%) and face shields (60.6%). Lower compliance was observed for gowns (53.9%), head covers (52.6%), coveralls (51.0%), and goggles/glasses (46.1%), with the latter representing the least frequently used PPE item.

Regarding knowledge of the WHO-recommended number of hand hygiene moments, only 41 (10.6%) participants correctly identified the recommended five moments. By contrast, 142 (36.8%) participants reported that they did not know the recommended number, and 120 (31.1%), 35 (9.1%), and 48 (12.4%) participants incorrectly reported three, four, and six moments, respectively.

### Perception of HCWs regarding COVID-19

Figure 5 illustrates the overall perception of HCWs toward COVID-19. Overall, only 31 (8.0%) participants demonstrated a positive perception, whereas the vast majority, 355 (92.0%), exhibited a negative perception.

**Figure 5:**
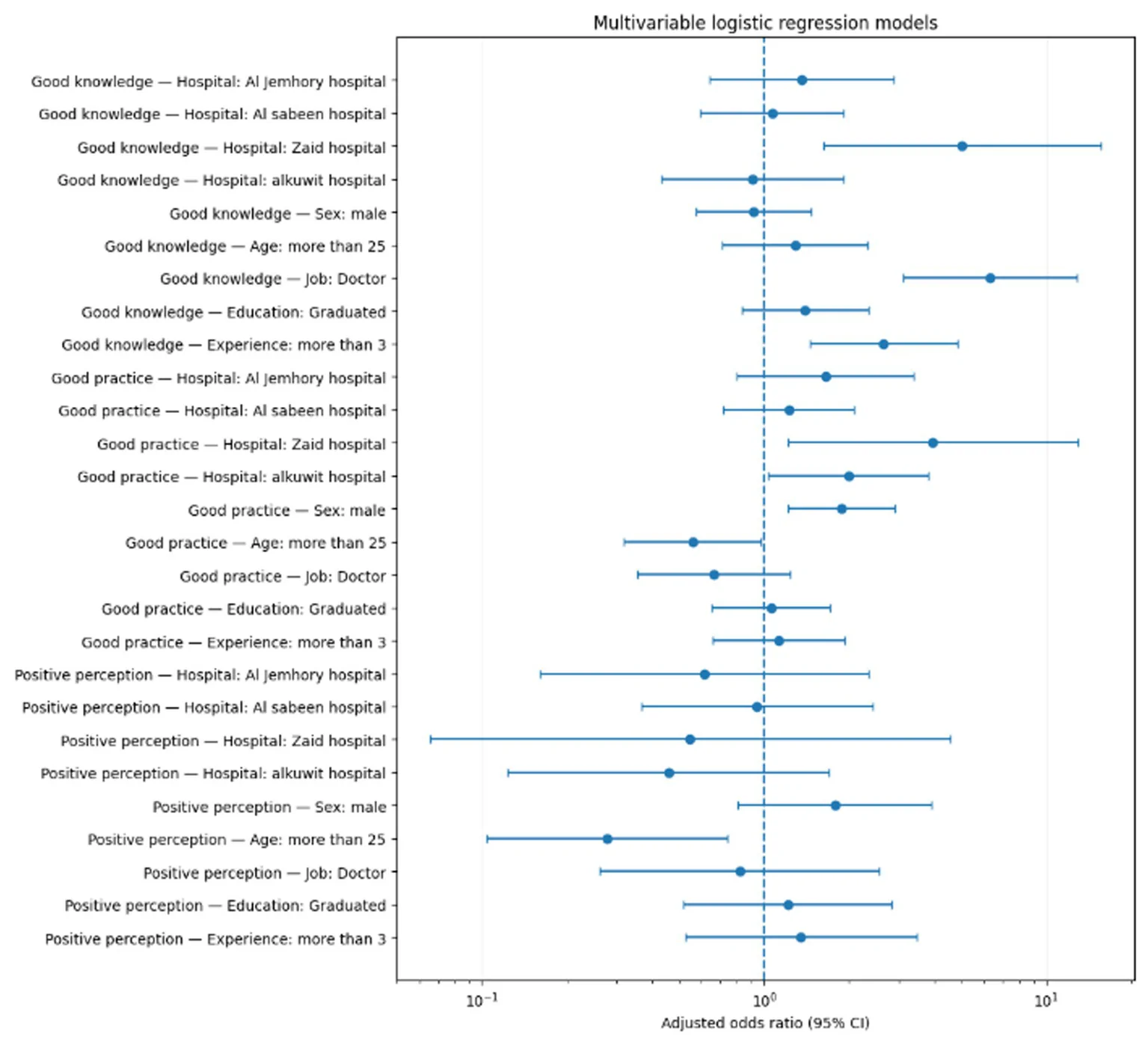
Forest plot of adjusted odds ratios for factors independently associated with good knowledge, good practice, and positive perception among healthcare workers.

As shown in Table 5, HCWs’ perceptions varied across different aspects of the COVID-19 pandemic. Most participants recognized that COVID-19 could affect individuals of all age groups and indicated their willingness to undergo isolation in healthcare facilities if infected. Although misconceptions remained regarding the availability of an effective cure for COVID-19, most respondents acknowledged the importance of quarantine, participation in COVID-19-related training programs, and adherence to social distancing measures.

**Table 5:** Summary of healthcare workers’ attitudes toward COVID-19.

Attitude items	Positive *n* (%)	Neutral *n* (%)	Negative *n* (%)	Mean ± (SD)
If the COVID-19 virus spread to the country, it will infect all ages	138 (35.8)	133 (34.5)	47 (12.2)	2.13 ± (1.12)
I will accept isolation in health facilities if I catch corona virus	164 (42.5)	138 (35.8)	32 (8.3)	1.99 ± (1.17)
I think there is a cure for the virus available on the market	36 (9.3)	55 (14.2)	96 (24.9)	3.36 ± (1.19)
I could go into quarantine with my family if asked	155 (40.2)	130 (33.7)	49 (12.7)	2.04 ± (1.14)
I am willing to participate in trainings for corona virus	207 (53.6)	115 (29.8)	47 (12.2)	1.68 ± (0.89)
I think social estrangement is necessary to limit the spread of the virus	242 (62.7)	103 (26.7)	30 (7.8)	1.52 ± (0.80)
I think traveling to a virus-free zone is an effective means to reduce infection	81 (21.0)	80 (20.7)	84 (21.8)	2.84 ± (1.31)
I think all patients are recovering from infection with COVID-19	44 (11.4)	97 (25.1)	113 (29.3)	2.90 ± (1.08)
I will stay in my area at the time of the COVID-19 pandemic	151 (39.1)	149 (38.6)	48 (12.4)	1.97 ± (1.05)
I will participate in programs related to the treatment and care of those infected with COVID-19	187 (48.4)	130 (33.7)	43 (11.1)	1.78 ± (0.95)
I think Yemen can win the fight against COVID-19	156 (40.4)	96 (24.9)	84 (21.8)	2.13 ± (1.19)
I will provide advice to friends and family about preventing COVID-19	258 (66.8)	106 (27.5)	19 (4.9)	1.40 ± (0.65)
I think the media can play a big role in reducing the infection	227 (58.8)	107 (27.7)	27 (7.0)	1.64 ± (0.97)
I will be afraid if someone in my residential area or around my work says he is sick with COVID-19	85 (22.0)	114 (29.5)	62 (16.1)	2.69 ± (1.32)
I may resign from my work in fear of being exposed to COVID-19	45 (11.7)	27 (7.0)	51 (13.2)	3.73 ± (1.32)

Perceptions regarding the likelihood of recovery from COVID-19 were not consistent, with a significant percentage of HCWs expressing neutral opinions. However, many HCWs reported their willingness to remain in their communities during the pandemic, participate in patient care programs, and contribute to national efforts aimed at controlling the spread of COVID-19.

Furthermore, most participants indicated that they would encourage their family members and friends to adopt preventive measures against COVID-19 and recognized the important role of media in increasing public awareness. Although concerns about occupational exposure to infection were evident, only a small proportion of HCWs reported that such concerns would make them consider resigning from their jobs (Table 5).

### Association between demographic characteristics and HCWs’ knowledge

A statistically significant association was found between the overall level of knowledge regarding COVID-19 and age, occupation, and years of experience (all *p* < 0.001). By contrast, no significant association was observed between knowledge level and sex (*p* = 0.411), as shown in Table 6.

**Table 6:** Association between demographic characteristics and healthcare workers’ knowledge toward COVID-19.

Characteristics	Category	Poor knowledge *n* (%)	Good knowledge *n* (%)	Total *n* (%)	*χ* ^2^	OR (95% CI)	*p* value
Sex	Male	104 (58.4%)	74 (41.6%)	178 (100%)	0.67	0.84 (0.56–1.27)	0.411
	Female	130 (62.5%)	78 (37.5%)	208 (100%)			
Total		234 (60.6%)	152 (39.4%)	386 (100%)			
Age groups (years)	˃25	115 (50.9%)	111 (49.1%)	226 (100%)	21.36	0.36 (0.23–0.55)	<0.001
	20–25	119 (74.4%)	41 (25.6%)	160 (100%)			
Total		234 (60.6%)	152 (39.4%)	386 (100%)			
Occupation/profession	Doctor	20 (8.5%)	55 (36.2%)	75 (19.4%)	44.9	0.17 (0.09–0.29)	<0.001
	Other HCWs	214 (91.5%)	97 (63.8%)	311 (80.6%)			
Total		234 (100%)	152 (100%)	386 (100%)			
Work experience (years)	˃3	103 (52.0%)	95 (48.0%)	198 (100%)	12.6	0.47 (0.31–0.72)	<0.001
	1–3	131 (69.7%)	57 (30.3%)	188 (100%)			
Total		234 (60.6%)	152 (39.4%)	386 (100%)			
Educational level	Graduate	93 (48.9%)	97 (51.10%)	190 (100%)	21.36	0.37 (0.24–0.57)	<0.001
	Undergraduate	141 (71.90%)	55 (28.10%)	196 (100%)			
Total		234 (60.6%)	152 (39.4%)	386 (100%)			

Data are presented as *n* (%). *χ*^2^, chi-square test; OR, odds ratio; CI, confidence interval. Statistical significance was considered at *p* < 0.05.

### Association between demographic characteristics and HCWs’ practice

Table 7 presents the association between demographic characteristics and HCWs’ practice toward COVID-19. A statistically significant association was observed between the level of practice and sex (*p* = 0.020), age (*p* = 0.020), and professional category (*p* = 0.030). By contrast, no statistically significant associations were found between practice level and educational level (*p* = 0.190) or years of work experience (*p* = 0.440).

**Table 7:** Association between demographic characteristics and healthcare workers’ practice toward COVID-19.

Characteristic	Category	Bad practice *n* (%)	Good practice *n* (%)	Total	*χ* ^2^	OR (95% CI)	*p*
Sex	Male	68 (38.20%)	110 (61.80%)	178 (100%)	5.40	0.62 (0.41–0.93)	0.020*
	Female	104 (50.0%)	104 (50.0%)	208 (100%)			
Total		172 (44.60%)	214 (55.4.00%)	(100%) 386			
Age groups (years)	˃25	112 (49.60%)	114 (50.40%)	226 (100%)	5.51	1.61 (1.05–2.47)	0.020*
	20–25	60 (37.5%)	100 (62.5%)	160 (100%)			
Total		172 (44.6%)	214 (55.4%)	(100%) 386			
Educational level	Graduate	91 (47.90%)	99 (52.10%)	190 (100%)	1.68	1.31 (0.87–1.95)	0.190
	Undergraduate	81 (41.30%)	115 (58.70)	196 (100%)			
Total		172 (44.60%)	214 (55.4%)	(100%) 386			
Occupation/profession	Doctor	42 (24.4%)	33 (15.4%)	75 (19.4%)	4.93	1.77 (1.07–2.95)	0.030*
	Other HCWs	130 (75.6%)	181 (84.6%)	214 (100%)			
Total		172 (100%)	214 (100%)	386 (100%)			
Work experience (years)	˃3	92 (46.50%)	106 (53.5%)	198 (100%)	0.60	1.17 (0.78–1.75)	0.440
	1–3	80 (42.60%)	108 (57.40%)	188 (100%)			
Total		172 (44.6%)	214 (55.40%)	386 (100%)			

Data are presented as *n* (%). *χ*^2^, chi-square test; OR, odds ratio; CI, confidence interval. *p* < 0.05 indicates statistical significance. Bold values indicate statistically significant results (*P* < 0.05).

### Association between demographic characteristics and HCWs’ perception

Table 8 summarizes the association between demographic characteristics and HCWs’ perception of COVID-19. A statistically significant association was observed only between age and perception level (*p* = 0.020). No statistically significant associations were found between perception level and sex (*p* = 0.310), educational level (*p* = 0.920), years of work experience (*p* = 0.480), and professional category (*p* = 0.630).

**Table 8:** Association between demographic characteristics and healthcare workers’ perception toward COVID-19.

Characteristic	Category	Positive perception *n* (%)	Negative perception *n* (%)	Total *n* (%)	*χ* ^2^	OR (95% CI)	*p* value
Sex	Male	17 (9.60%)	161 (90.40%)	178 (100%)	1.03	1.46 (0.70–3.06)	0.312
	Female	14 (6.70%)	194 (93.30%)	208 (100%)			
Total		31 (8.00%)	355 (92.00%)	386 (100%)			
Age groups (years)	˃25	12 (5.30%)	214 (94.70%)	226 (100%)	5.477	0.42 (0.20–0.88)	0.020*
	20–25	19 (11.9%)	141 (88.10%)	160 (100%)			
Total		31 (8.00%)	355 (92.00%)	386 (100%)			
Educational level	Graduate	15 (7.9%)	175 (92.1%)	190 (100%)	0.01	0.97 (0.46–2.01)	0.923
	Undergraduate	16 (8.2%)	180 (91.8)	196 (100%)			
Total		31 (8.00%)	355 (92.00%)	386 (100%)			
Occupation/profession	Doctor	5 (16.1%)	70 (19.7%)	75 (100%)	0.23	0.78 (0.29–2.11)	0.633
	Other HWs	26 (83.9%)	285 (80.3%)	355 (100%)			
Total		31 (100%)	355 (100%)	386 (100%)			
Work experience (years)	˃3	14 (7.1%)	184 (92.9%)	198 (100%)	0.51	0.77 (0.37–1.60)	0.486
	1–3	17 (9.00%)	171 (91.00%)	188 (100%)			
Total		31 (8.0%)	355 (92.00%)	386 (100%)			

Data are presented as *n* (%). *χ*^2^, chi-square test; OR, odds ratio; CI, confidence interval. Statistical significance was defined as *p* < 0.05. Bold values indicate statistically significant results (*p* < 0.05).

To account for the risk of false-positive findings arising from multiple comparisons, Benjamini–Hochberg (BH) correction was applied to the 15 chi-square tests examining associations between five demographic/professional characteristics and the three study outcomes (knowledge, practice, and perception). After BH adjustment, age, occupation, educational level, and work experience remained significantly associated with knowledge (all adjusted *p* < 0.0038). For practice, sex and age remained statistically significant (adjusted *p* = 0.0429 for both), whereas the association with professional occupation was no longer significant after correction (adjusted *p* = 0.0563). For perception, age remained significantly associated with the outcome (adjusted *p* = 0.0429), while sex, occupation, educational level, and work experience were not significant after adjustment. These findings indicate that most of the principal associations were robust to correction for multiple testing, although the originally significant association between occupation and practice should be interpreted cautiously (Table 9).

**Table 9:** Environmental factors related to COVID-19 preparedness (*N* = 5).

Assessed item	Yes, *n* (%)	No, *n* (%)
Are there negative-pressure infection isolation rooms?	0 (0.0)	5 (100.0)
Is alcohol-based hand rub easily available (i.e., at the point of care) for hand hygiene within the healthcare facility?	0 (0.0)	5 (100.0)
Does the healthcare facility adopt a universal masking policy for all HWs?	1 (20.0)	4 (80.0)
Are soap and water available for hand hygiene within the healthcare facility?	2 (40.0)	3 (60.0)
Is a medical mask fitted to patients with suspected SARS-CoV-2 infection upon arrival at the healthcare facility?	2 (40.0)	3 (60.0)
Does the healthcare facility have a well-equipped triage station at the entrance?	3 (60.0)	2 (40.0)
Is PPE available in sufficient quantity in the healthcare facility?	3 (60.0)	2 (40.0)
Does the healthcare facility have PPE available?	4 (80.0)	1 (20.0)
Is there is a suitable distance between each bed?	4 (80.0)	1 (20.0)
Is there adequate ventilation in the isolation rooms?	4 (80.0)	1 (20.0)

### Environmental factors related to COVID-19 preparedness

The environmental preparedness of the participating hospitals was assessed using an observational checklist, and the findings are presented in Table 10. Four of the five hospitals had adequate room ventilation, maintained appropriate spacing between patient beds, and ensured the availability of PPE. Three hospitals had an adequate supply of PPE and a well-equipped triage station at the hospital entrance. Only two hospitals ensured that patients with suspected COVID-19 wore face masks upon arrival and provided soap and water for hand hygiene. Universal masking policies were implemented in only one hospital. Notably, none of the participating hospitals had alcohol-based hand rubs readily available or negative-pressure isolation rooms for infection control.

**Table 10:** Multivariable predictors of good knowledge, good practice, and positive perception among healthcare workers.

Outcome	Predictor	Adjusted OR	95% CI	*p*
Good knowledge	Zaid vs. Al-Thawrah	5.03	1.62–15.60	0.005
	Doctor vs. other HCWs	6.32	3.11–12.84	<0.001
	>3 years vs. 1–3 years	2.65	1.45–4.83	0.001
Good practice	Zaid vs. Al-Thawrah	3.96	1.21–12.92	0.023
	Al-Kuwait vs. Al-Thawrah	1.99	1.04–3.83	0.038
	Male vs. female	1.88	1.22–2.91	0.004
	>25 vs. 20–25 years	0.56	0.32–0.98	0.041
Positive perception	>25 vs. 20–25 years	0.28	0.10–0.74	0.011

Model	LR *χ*^2^	Df	Overall *p*	McFadden *R*^2^
Good knowledge	78.04	9	**<0.001**	0.151
Good practice	26.96	9	**0.001**	0.051
Positive perception	10.40	9	0.319	0.048

### Multivariable logistic regression analysis

Multivariable logistic regression analysis identified several independent predictors of the study outcomes (Table 11). The overall model for good knowledge was statistically significant (likelihood-ratio *χ*^2^ = 78.04, df = 9, *p* < 0.001; McFadden’s *R*^2^ = 0.151). HCWs working at Zaid Hospital had significantly higher odds of demonstrating good knowledge than those working at Al-Thawrah Hospital (aOR = 5.03, 95% CI: 1.62–15.60, *p* = 0.005). Doctors also had substantially higher odds of good knowledge than other HCWs (aOR = 6.32, 95% CI: 3.11–12.84, *p* < 0.001). In addition, HCWs with more than 3 years of professional experience had higher odds of good knowledge than those with 1–3 years of experience (aOR = 2.65, 95% CI: 1.45–4.83, *p* = 0.001).

**Table 11:** Sensitivity analysis of alternative classification thresholds.

Domain	Original cut-off	60% cut-off	75% cut-off
Knowledge (46 points)	≥33/46 → **152 (39.4%)**	≥28/46 → **304 (78.8%)**	≥35/46 → **99 (25.6%)**
Practice (14 points)	≥10/14 → **182 (47.2%)**	≥9/14 → **214 (55.4%)**	≥11/14 → **151 (39.1%)**
Perception (75 points)	≥51/75 → **31 (8.0%)**	≥45/75 → **17 (4.4%)**	≥57/75 → **0 (0.0%)**

The original cut-offs were prespecified in the study scoring protocol. For knowledge and practice, the primary threshold was ≥70% of the maximum score. For perception, the prespecified threshold was ≥51/75. Alternative thresholds corresponding to 60 and 75% of the maximum possible score were evaluated in sensitivity analyses. Because no independent external criterion or gold-standard outcome was available, ROC-based empirical threshold derivation and sensitivity/specificity analyses were not performed. Bold values indicate statistically significant results (*P* < 0.05).

The multivariable model for good practice was also statistically significant (likelihood-ratio *χ*^2^ = 26.96, df = 9, *p* = 0.001; McFadden’s *R*^2^ = 0.051). Compared with HCWs at Al-Thawrah Hospital, those working at Zaid Hospital had higher odds of good practice (aOR = 3.96, 95% CI: 1.21–12.92, *p* = 0.023), as did those working at Al-Kuwait Hospital (aOR = 1.99, 95% CI: 1.04–3.83, *p* = 0.038). Male HCWs had higher odds of good practice than female HCWs (aOR = 1.88, 95% CI: 1.22–2.91, *p* = 0.004). Conversely, HCWs aged >25 years had lower odds of good practice than those aged 20–25 years (aOR = 0.56, 95% CI: 0.32–0.98, *p* = 0.041).

The overall model for positive perception was not statistically significant (likelihood-ratio *χ*^2^ = 10.40, df = 9, *p* = 0.319; McFadden’s *R*^2^ = 0.048). Nevertheless, age was independently associated with perception: HCWs aged >25 years had significantly lower odds of positive perception than those aged 20–25 years (aOR = 0.28, 95% CI: 0.10–0.74, *p* = 0.011).

### Sensitivity analysis of classification thresholds

Sensitivity analyses were performed to assess the robustness of the prespecified dichotomization thresholds using alternative cut-points corresponding to 60 and 75% of the maximum possible score (Table 12). For knowledge, 304 participants (78.8%) were classified as having good knowledge at the 60% threshold (≥28/46), compared with 152 (39.4%) at the original prespecified threshold of ≥33/46 (70%) and 99 (25.6%) at the 75% threshold (≥35/46). For practice, 214 participants (55.4%) met the 60% threshold (≥9/14), compared with 182 (47.2%) at the original prespecified threshold of ≥10/14 (70%) and 151 (39.1%) at the 75% threshold (≥11/14).

**Table 12:** Benjamini–Hochberg-adjusted *p*-values for associations between demographic characteristics and knowledge, practice, and perception.

Outcome	Demographic factor	Original *p*-value	BH-adjusted *p*-value	Significance after BH correction
Knowledge	Age	<0.001	<0.0038	Significant
	Sex	0.411	0.550	Not significant
	Occupation	<0.001	<0.0038	Significant
	Educational level	<0.001	<0.0038	Significant
	Work experience	<0.001	<0.0038	Significant
Practice	Sex	0.020	0.0429	Significant
	Age	0.020	0.0429	Significant
	Occupation	0.030	0.0563	Not significant
	Educational level	0.190	0.3167	Not significant
	Work experience	0.440	0.550	Not significant
Perception	Age	0.020	0.0429	Significant
	Sex	0.310	0.4650	Not significant
	Educational level	0.920	0.9200	Not significant
	Work experience	0.480	0.5538	Not significant
	Occupation	0.630	0.6750	Not significant

For perception, 17 participants (4.4%) met the 60% threshold (≥45/75), compared with 31 participants (8.0%) at the original prespecified threshold of ≥51/75 and none (0.0%) at the 75% threshold (≥57/75). For reference, applying a strict 70% threshold to the perception scale (≥53/75) also resulted in 0 participants being classified as having a positive perception.

Overall, the proportion of participants classified as having good or positive outcomes varied substantially across alternative cut-points, particularly for knowledge and perception, indicating sensitivity of the classification to the selected threshold.

ROC-based empirical threshold derivation and diagnostic sensitivity/specificity analyses were not performed because no independent external criterion or gold-standard outcome was available in the dataset.

## Discussion

This study provides one of the few comprehensive evaluations of COVID-19 IPC measures among HCWs in public hospitals in Yemen, integrating their knowledge, practices, and perceptions KAP direct observations of institutional preparedness. To the authors’ knowledge, this study is among the first studies in the Yemeni context to combine individual-level behavioral assessments with facility-level environmental observations, thereby providing a more comprehensive understanding of multiple factors influencing IPC implementation.

Overall, the findings reveal substantial deficiencies in COVID-19 preparedness despite more than 3 years of experience managing the pandemic. Fewer than half of the participating HCWs demonstrated adequate overall knowledge, preventive practices were only moderate, and positive perceptions toward COVID-19 prevention remained uncommon. The observational assessment identified considerable shortcomings in healthcare facility preparedness, including inadequate application of masking policies, limited availability of alcohol-based hand hygiene services, and the complete absence of isolation rooms. These findings indicate that important gaps persist at the individual and institutional levels.

HCWs represent the cornerstone of IPC because they are continuously exposed to respiratory pathogens in high-risk clinical environments. Therefore, effective IPC depends not only on HCWs’ knowledge and adherence to evidence-based preventive practices but also on the availability of adequate institutional resources, supportive organizational policies, and a resilient healthcare infrastructure capable of responding to emerging infectious diseases (24). In resource-constrained and conflict-affected health systems such as Yemen, deficiencies across these interconnected domains may considerably increase the risk of occupational exposure, healthcare-associated transmission, and disruption of essential healthcare services during future infectious disease outbreaks.

### Knowledge on COVID-19 among HCWs

Fewer than half of the participating HCWs achieved an adequate overall level of knowledge regarding COVID-19, with only 39.7% meeting the predefined threshold for good knowledge. While participants demonstrated relatively satisfactory knowledge of the disease concept (61.9%) and treatment recommendations (54.5%), considerable deficiencies were identified in other knowledge domains, particularly the modes of SARS-CoV-2 transmission, where only 7.5% demonstrated adequate knowledge. Overall, these findings suggest that HCWs possessed a better understanding of the clinical aspects of COVID-19 than of the infection prevention and transmission principles required for the effective application of IPC measures.

Among the assessed knowledge domains, the concept of COVID-19 showed the highest rate of correct responses. Most participants correctly identified COVID-19 as an infectious disease and recognized the importance of isolating suspected cases. This relatively reasonable performance may reflect extensive public health campaigns, continuous media coverage, and the widespread dissemination of WHO recommendations throughout the pandemic. Similar findings were reported in a study conducted earlier in Yemen, in which HCWs demonstrated good awareness of the basic characteristics of COVID-19 despite working in a conflict-affected healthcare system (21). Systematic evidence from low- and middle-income countries indicates that HCWs generally possess stronger conceptual knowledge than operational IPC knowledge (16). However, misconceptions regarding disease severity and clinical classification persisted among a substantial proportion of participants, suggesting that general awareness alone is insufficient to ensure comprehensive understanding of evolving clinical definitions.

In contrast to the relatively satisfactory conceptual knowledge, the participants demonstrated remarkably poor knowledge regarding the transmission of SARS-CoV-2. Although most HCWs correctly identified respiratory droplets as the primary route of transmission, misconceptions regarding airborne, waterborne, bloodborne, foodborne, and fomite transmissions remained. These findings likely reflect the rapidly evolving scientific evidence and frequent revisions of recommendations during the early stages of the pandemic, which may have created uncertainty among frontline HCWs. Still, inconsistent dissemination of updated IPC guidelines within healthcare institutions may have contributed to the persistence of these misconceptions.

The findings are partially consistent with those reported earlier in Yemen (21), that is, although HCWs demonstrated generally satisfactory overall knowledge of COVID-19, important gaps remained in specific areas related to disease preparedness and infection control. The variances between the two studies may be explained by differences in the assessment tools, scoring criteria, healthcare settings, and the evolving nature of scientific evidence regarding SARS-CoV-2 transmission throughout the pandemic. This study evaluated detailed and comprehensive transmission dynamics routes such as airborne, fomite, foodborne, and bloodborne.

Moreover, prolonged healthcare system fragmentation, limited access to continuing professional education, and inconsistent implementation of national IPC policies may have further contributed to these knowledge gaps among Yemeni HCWs. Previous reports have shown that years of armed conflict have substantially weakened Yemen’s healthcare infrastructure, reducing opportunities for continuous training and limiting institutional capacity to implement updated infection prevention recommendations (19).

Similar deficiencies have been documented in recent systematic reviews, which reported persistent gaps in HCWs’ understanding of SARS-CoV-2 transmission despite generally acceptable awareness of the disease itself. These reviews concluded that inadequate knowledge of transmission pathways is associated with inconsistent adherence to PPE recommendations, suboptimal implementation of IPC measures, and an increased risk of occupational and healthcare-associated transmission (16, 25).

Knowledge regarding the clinical manifestations of COVID-19 varied considerably across symptom categories. HCWs correctly recognized the classic respiratory symptoms, including fever, cough, altered taste, malaise, headache, and myalgia or muscle aches. This discrepancy is understandable because public health messaging, clinical guidelines, and HCW training during the early stages of the pandemic primarily emphasized respiratory symptoms and severe disease, whereas awareness of extrapulmonary manifestations expanded only as additional clinical evidence accumulated (18, 23, 26).

The study findings are broadly consistent with those reported in a previous Yemeni study, which demonstrated satisfactory knowledge on the common clinical features of COVID-19 among HCWs but identified important gaps in disease preparedness and several aspects of COVID-19-related knowledge. Though that study did not specifically evaluate recognition of atypical appearances, both studies suggest that HCWs’ knowledge was stronger for respiratory symptoms than for less often encountered clinical appearances (21).

These findings are also supported by international evidence. A systematic review reported that HCWs consistently demonstrated better recognition of common COVID-19 symptoms than atypical or extrapulmonary manifestations, reflecting the emphasis of early IPC education on respiratory disease (16). Similarly, recent reviews have shown that recognition of non-respiratory manifestations, including dermatological, neurological, gastrointestinal, and ophthalmological symptoms, improved only after accumulating evidence established the multisystem nature of SARS-CoV-2 infection (16, 27).

Failure to identify atypical clinical signs may delay early diagnosis, patient isolation, diagnostic testing, and application of appropriate prevention and control measures, thereby increasing the risk of HCW-associated transmission. This concern is mainly related in resource-constrained and conflict-affected healthcare systems, such as Yemen, where inadequate diagnostic capacity, shortages of healthcare resources, and fragmented health services make early clinical recognition an essential component of outbreak response (19). So, continuous development programs should extend beyond the recognition of classic respiratory symptoms to include atypical and emerging clinical signs, ensuring that HCWs remain ready to identify evolving presentations of COVID-19 and future emerging infectious diseases.

In relation to knowledge of transmission and clinical manifestations, most HCWs in the present study correctly recognized hand hygiene and personal protective measures as effective strategies to prevent COVID-19 transmission (95%). Appropriate use of PPE, including respirators, gloves, gowns, and eye protection, was generally high, with correct response rates ranging from 88.1 to 97.9%. These findings suggest that repeated public health messaging and IPC campaigns during the pandemic successfully reinforced awareness of fundamental preventive interventions among HCWs. Similar remarks have been reported globally, where HCWs frequently verified better knowledge of preventive strategies than other domains of COVID-19 knowledge, reflecting the extensive emphasis placed on hand hygiene, PPE, sharp safety, respiratory hygiene, injection safety, sterilization of instruments and devices, and environmental cleanliness (16, 28).

However, despite this relatively favorable level of preventive knowledge, some uncertainty persisted regarding specific recommendations, mainly mask use among asymptomatic individuals and triage-related precautions (29, 30). This discrepancy may reflect the dynamic nature of COVID-19 situations, which evolved considerably as evidence regarding viral transmission, asymptomatic infection, and aerosol-generating procedures accumulated. Frequent changes in international guidance, combined with inconsistent institutional dissemination of updated IPC protocols, may have contributed to confusion among frontline HCWs (3). Previous studies have similarly emphasized that maintaining HCW competency during emerging infectious disease outbreaks requires continuous education rather than one-time training interventions, particularly when recommendations change rapidly (16, 17, 31).

The fairly satisfactory knowledge of PPE-related prevention measures should be interpreted cautiously because knowledge does not always translate into reliable clinical practice. Although PPE knowledge was high, actual adherence to several PPE items were suboptimal, particularly for gowns, eye protection, and coveralls. This finding highlights the well-recognized gap between awareness and implementation of IPC measures. Previous systematic reviews have verified that PPE compliance among HCWs is influenced by factors such as the availability of equipment, comfort, workload, perceived occupational risk, and workplace safety (17, 32, 33).

In the context of Yemen, these findings are particularly relevant because prolonged conflict and healthcare system fragmentation have negatively affected the availability of infection prevention resources, continuous training opportunities, and implementation of standardized IPC policies. Therefore, improving preventive practices requires not only strengthening HCWs’ knowledge but also addressing organizational and structural barriers through reliable PPE supply chains, regular competency-based IPC training, effective supervision, and updated institutional guidelines (3, 18, 19, 34). A sustainable prevention strategy should integrate individual behavioral change with supportive healthcare system interventions to enhance preparedness for future respiratory infectious disease outbreaks.

The participants in this study demonstrated relatively satisfactory knowledge regarding supportive management of COVID-19, particularly in relation to patient screening at the first point of healthcare contact, symptomatic management, nutritional support, and psychosocial care. This finding may reflect the extensive dissemination of WHO clinical management guidelines during the pandemic, together with the progressive accumulation of clinical experience among HCWs through successive waves of SARS-CoV-2 infection. Similar observations have been reported in previous studies, where HCWs showed better knowledge of basic COVID-19 management principles than the more complex aspects of clinical decision-making, suggesting that repeated exposure to standardized recommendations can improve awareness of essential treatment strategies (3, 22, 35).

The high level of recognition of screening and early assessment measures observed in this study is particularly important because timely identification and clinical evaluation of suspected cases represent critical components of outbreak control and patient management. WHO recommendations have emphasized the importance of early triage, severity assessment, and appropriate referral pathways to prevent clinical deterioration and reduce healthcare-associated transmission (3, 23, 36, 37). In resource-limited settings where diagnostic capacity and critical care resources may be constrained, effective screening and early clinical assessment become more essential components of pandemic preparedness.

Despite satisfactory knowledge of several supportive care measures, important gaps were identified regarding the management of mild and moderate COVID-19 and fluid management in severe cases. These deficiencies may indicate limited understanding of evolving clinical management recommendations, particularly as treatment strategies changed substantially throughout the pandemic with emerging evidence regarding disease severity, oxygen requirements, antiviral therapies, and supportive interventions. Previous research has highlighted that HCWs in low- and middle-income countries frequently experience challenges in maintaining updated knowledge during rapidly evolving outbreaks due to limited access to continuing medical education, inconsistent dissemination of updated guidelines, and healthcare system constraints (16, 17, 37).

The observed uncertainty regarding fluid management in severe COVID-19 cases is clinically relevant because inappropriate fluid administration may contribute to worsening respiratory compromise, particularly among patients with severe pneumonia or acute respiratory distress syndrome. Current clinical guidelines recommend cautious fluid management in critically ill patients to avoid fluid overload and associated adverse outcomes (13). Therefore, targeted competency-based training focusing on severity assessment, supportive management principles, and updates in evidence-based treatment protocols remains essential to improve HCWs’ preparedness for future respiratory infectious disease emergencies.

In the context of Yemen, maintaining adequate clinical knowledge represents a considerable challenge due to prolonged conflict, fragmentation of healthcare services, shortages of trained personnel, and limited opportunities for continuous professional development. These systemic challenges have weakened healthcare capacity and restricted the implementation of updated clinical guidelines during health emergencies (19). Consequently, improving treatment-related preparedness requires not only individual knowledge enhancement but also sustainable institutional strategies, including regular clinical training programs, access to updated WHO recommendations, and strengthening healthcare system resilience.

### COVID-19 IPC practices of HCWs

This study demonstrated that 55.4% of HCWs exhibited good IPC practice toward COVID-19, indicating a moderate level of adherence to recommended preventive measures and highlighting a persistent gap in the consistent implementation of evidence-based IPC practices among HCWs, particularly within resource-constrained healthcare systems. Comparable levels of preventive practice have been reported across several countries in the Middle East and Africa, where HCWs frequently faced shortages of infection control resources, inconsistent access to updated IPC training, and high clinical workloads throughout the pandemic (21, 38–40). These similarities suggest that the challenges observed in the present study reflect broader systemic constraints affecting healthcare systems across low- and middle-income settings rather than isolated institutional deficiencies.

Overall, preventive practice exceeded the overall knowledge level observed in this study, suggesting that HCWs’ behavior was influenced by not only theoretical knowledge but also institutional regulations, workplace culture, routine supervision, and mandatory infection control protocols. Adherence to IPC measures is reinforced through organizational policies and continuous monitoring, enabling acceptable compliance even when comprehensive theoretical understanding is limited. This observation is consistent with behavioral models of infection prevention, which recognize organizational support, perceived occupational risk, and institutional safety culture as major causes of IPC compliance alongside knowledge (41).

Hand hygiene represented one of the strongest aspects of preventive practice, with 77.2% of the participants reporting appropriate hand hygiene behavior. This high level of compliance likely reflects the extensive emphasis placed on hand hygiene throughout the pandemic because it remains one of the most effective and cost-efficient strategies for preventing HCW-associated infections and limiting viral transmission (16). Similar progresses in hand hygiene compliance have been reported among HCWs in Saudi Arabia, Ethiopia, and Uganda following intensified infection prevention campaigns during the pandemic, although deficiencies in knowledge of evidence-based hand hygiene recommendations remained common (16, 40, 42).

Although most of the participants routinely performed hand hygiene, only 10.6% correctly identified WHO’s “Five Moments for Hand Hygiene.” This finding suggests that hand hygiene may have become a habitual workplace behavior driven by institutional expectations rather than a practice supported by a comprehensive understanding of evidence-based IPC principles. Similar discrepancies between practical compliance and theoretical knowledge have been reported among HCWs in several low- and middle-income countries, emphasizing the importance of competency-based educational programs that strengthen conceptual understanding and practical application (16, 43).

By contrast, the adherence to PPE recommendations was inconsistent across types of protective equipment. Gloves and surgical masks demonstrated the highest utilization rates, whereas compliance with gowns, eye protection, coveralls, and head covers remained low. While inadequate PPE availability likely contributed to these findings, factors such as inconsistent institutional enforcement of IPC policies, discomfort associated with prolonged PPE use, heavy clinical workloads, staff fatigue, and variations in perceived occupational risk may have influenced adherence. Similar patterns have been documented in healthcare facilities across Yemen and other low-resource settings in the Middle East and sub-Saharan Africa, where shortages of PPE, disrupted supply chains, and limited institutional preparedness substantially affected HCWs’ ability to fully comply with recommended IPC measures (17, 44). These findings indicate that inadequate PPE compliance during the pandemic was driven by not only individual behavior but also structural and organizational constraints.

Collectively, these findings suggest that improving HCWs’ knowledge alone is unlikely to achieve sustained improvements in infection prevention practices. Instead, effective IPC requires a multifaceted strategy that integrates continuous professional education, competency-based training, reliable availability of PPE, supportive institutional leadership, regular supervision, and organizational cultures that prioritize patients’ and HCWs’ safety. Such comprehensive interventions are particularly critical in fragile healthcare systems, including Yemen and other conflict-affected countries, where resource constraints, workforce shortages, and disruptions to healthcare infrastructure may further hinder adherence to evidence-based infection prevention practices.

### Perception of HCWs regarding COVID-19

Despite generally acceptable responses to several individual perception items, the overall perception score was predominantly negative, with only 8.0% of HCWs demonstrating a positive perception toward COVID-19 IPC. This discrepancy suggests that although participants agreed with specific preventive measures, their overall confidence, risk perception, and attitudes toward COVID-19 prevention remained suboptimal. Similar findings have been reported among HCWs in low- and middle-income countries, where fear of infection, inadequate institutional support, uncertainty regarding evolving guidelines, and prolonged occupational stress negatively influenced HCWs’ perceptions despite reasonable knowledge of preventive measures (16, 39, 45).

Interestingly, most participants expressed willingness to undergo isolation if infected, participate in training programs, adhere to social distancing measures, and educate family members about COVID-19 prevention. These findings indicate a generally positive attitude toward personal and community responsibility despite the low overall perception score. Such willingness may reflect HCWs’ professional commitment and recognition of their public health role during infectious disease outbreaks.

Conversely, misconceptions regarding the availability of an effective cure and uncertainty about recovery from COVID-19 remained common. This finding may reflect the rapidly evolving scientific evidence available during the pandemic and the inconsistent dissemination of updated recommendations within healthcare institutions. Moreover, prolonged exposure to pandemic-related stress, limited institutional preparedness, and working under resource-constrained conditions may have contributed to reduced confidence in healthcare systems and negatively affected overall perceptions.

### Association between demographic characteristics and knowledge, practice, and perception

This study demonstrated that HCWs’ knowledge was significantly associated with age, educational level, professional category, and years of experience [Table 4]. Older HCWs, those with higher educational qualifications, physicians, and participants with longer professional experience demonstrated significantly better knowledge. These findings are consistent with previous studies (21, 25, 38, 40) suggesting that accumulated clinical experience, postgraduate education, and greater exposure to continuing professional development contribute to improved understanding of infectious disease prevention and control.

Practice was significantly associated with sex, age, and professional category, with younger HCWs demonstrating better preventive practices despite having lower knowledge scores. This finding may indicate that recently trained HCWs are more likely to adhere to current IPC recommendations because of greater familiarity with updated infection control protocols and recent educational curricula emphasizing evidence-based practice.

Regarding perception, only age showed a statistically significant association. This finding recommends that perceptions toward COVID-19 are influenced by not only professional background but also personal practices, mental resilience, workplace culture, institutional support, and perceived occupational risk, which are factors that have consistently been identified in recent systematic reviews examining HCWs during the COVID-19 pandemic (46–49).

### Organizational and environmental factors

A substantial deficiency was found in institutional preparedness for COVID-19 IPC across the participating hospitals. None of the hospitals had negative-pressure isolation rooms or alcohol-based hand rub available at the point of care, and only one hospital had implemented a universal masking policy. Moreover, routine COVID-19 screening strategies for HCWs and COVID-19-specific IPC guidelines were absent across all participating hospitals. These findings indicate considerable structural limitations that likely reduced HCWs’ ability to consistently comply with recommended IPC measures.

Although adequate ventilation, appropriate patient bed spacing, and general PPE availability were observed in most hospitals, the availability of PPE in sufficient quantities remained inconsistent, and only a minority of hospitals ensured access to soap and water or provided face masks to patients upon arrival. These findings suggest that partial application of IPC infrastructure alone is insufficient to achieve ideal infection prevention without comprehensive institutional policies, continuous supply chains, and organizational commitment.

The organizational deficiencies identified in this study are consistent with previous evidence from conflict-affected and resource-limited healthcare systems, where shortages of essential supplies, fragmented health services, insufficient IPC governance, and inadequate training substantially compromise HCWs’ adherence to IPC measures (17, 19, 23, 44, 50–52).

Years of armed conflict have further weakened Yemen’s healthcare infrastructure, making sustained implementation of evidence-based IPC recommendations particularly challenging. Thus, strengthening institutional preparedness should be considered a priority alongside improving HCWs’ knowledge and behavior. Continuous IPC training, implementation of updated guidelines, reliable availability of PPE and hand hygiene resources, establishment of effective screening systems, and strengthened administrative support are essential to improve preparedness for future respiratory infectious disease outbreaks.

### Strengths and limitations

A major strength of this study is the inclusion of healthcare professionals from diverse occupational categories across five public hospitals, enabling a comprehensive assessment of knowledge, IPC practices, and perceptions and facilitating the identification of areas requiring targeted improvement. The use of stratified random sampling across hospitals and professional categories enhanced representation of the participating HCWs within the study setting. In addition, the use of an observational checklist alongside the self-administered questionnaire provided complementary information on institutional, environmental, and organizational IPC conditions. The study also incorporated several analytical procedures to assess the robustness of the findings, including multivariable logistic regression, Benjamini–Hochberg correction for multiple comparisons, sensitivity analyses using alternative dichotomization thresholds, and *post hoc* assessment of domain-specific internal consistency and item-level performance.

Nevertheless, several limitations should be considered. First, the cross-sectional design precludes establishing causal relationships between participants’ characteristics and their knowledge, practices, or perceptions. Associations identified in this study should therefore be interpreted as correlational rather than causal.

Second, reliance on a self-administered questionnaire may have introduced recall and social-desirability bias, potentially resulting in overestimation of reported IPC practices. Although an observational checklist was used to provide complementary information, it primarily assessed institutional, environmental, and organizational IPC conditions rather than systematically observing individual-level adherence to IPC behaviors. Therefore, these observations should not be considered a direct validation of participants’ self-reported practices.

Third, although stratified random sampling was used across hospitals and professional categories, the study was limited to five selected public hospitals in Sana’a City. Therefore, the findings may not be fully generalizable to HCWs in other hospitals, healthcare sectors, or geographical regions of Yemen. Because proportional allocation was incorporated into the sampling design, no sampling or post-stratification weights were applied in the analysis. Consequently, the findings reflect the observed study sample and may not precisely reproduce the distribution of HCWs across the broader healthcare workforce. In addition, potential nonresponse bias cannot be excluded. Questionnaires were distributed during both morning and evening shifts to improve coverage of HCWs with different work schedules; however, individual-level information on nonrespondents was unavailable. Therefore, systematic differences between respondents and eligible HCWs who declined participation or were unavailable cannot be ruled out, particularly with respect to workload, IPC training, professional responsibilities, knowledge, or practices.

Fourth, age and professional experience were categorized into two broad groups for the primary analyses. Although these predefined categories were applied consistently and sensitivity analyses were performed for the outcome classification thresholds, the dichotomization of age and experience may have resulted in loss of information and may have obscured potentially meaningful gradients within these variables. Accordingly, associations involving age and professional experience should be interpreted with caution, and future studies should examine these variables using more detailed, empirically justified categories or as continuous variables where appropriate.

Fifth, the evolving nature of COVID-19 evidence and IPC recommendations during the pandemic may have influenced HCWs’ knowledge, practices, and perceptions at the time of data collection. The findings should therefore be interpreted within the specific epidemiological and healthcare context of Yemen during September–November 2023.

Sixth, although multiple statistical comparisons were considered in the analysis, the relatively large number of demographic-outcome comparisons remains a potential source of false-positive findings. To address this issue, the Benjamini–Hochberg procedure was applied to the 15 chi-square tests examining associations between five demographic/professional characteristics and the three primary outcomes. Most of the principal associations remained statistically significant after correction; however, the initially significant association between professional category and practice did not remain significant after adjustment. Therefore, findings that were sensitive to multiplicity correction should be interpreted cautiously.

Seventh, although multivariable logistic regression was performed to adjust for several potential confounding variables, the models included hospital as a fixed effect rather than explicitly modelling the hierarchical structure of HCWs nested within hospitals. Consequently, potential within-hospital correlation may not have been fully accounted for, and residual clustering effects cannot be completely excluded. In addition, the relatively small number of hospitals (five) limits the feasibility and reliability of more complex multilevel modelling approaches. The multivariable models should therefore be interpreted as adjusted associations rather than definitive causal effects.

Eighth, the *post hoc* domain-specific reliability analyses indicated variable internal consistency across the predefined knowledge domains, with particularly low Cronbach’s alpha coefficients for the prevention and treatment domains. These domains were developed primarily as content domains rather than independently validated psychometric subscales; therefore, their alpha coefficients should be interpreted cautiously. Similarly, the transmission-domain item analysis identified substantial heterogeneity in item performance. These findings highlight areas for refinement of the questionnaire in future studies but do not by themselves establish inadequate content validity.

Ninth, sensitivity analyses demonstrated that the proportion of participants classified as having good knowledge, good practice, or positive perception varied substantially according to the selected dichotomization threshold, particularly for knowledge and perception. Because no independent external criterion or gold-standard outcome was available, ROC-based empirical threshold derivation and diagnostic sensitivity/specificity analyses could not be performed. The findings should therefore be interpreted in light of the prespecified primary thresholds and the observed sensitivity to alternative cut-points.

Finally, the observational checklist was study-specific and was not based primarily on the WHO infection prevention and control assessment framework (IPCAF), and formal inter-rater reliability was not assessed during the original data collection. Although observers received standardized instructions and the same assessment procedures were applied across hospitals, measurement variability in the observational component cannot be completely excluded.

Future studies should address these limitations through larger, multicenter investigations involving hospitals from different geographical regions and healthcare sectors to improve the generalizability of the findings. Longitudinal studies with repeated assessments would help evaluate changes in HCWs’ preparedness and determine the sustained effects of IPC training and institutional interventions. Structured and repeated direct observation of individual IPC behaviors, particularly hand hygiene and appropriate PPE use, would provide stronger triangulation of self-reported practices and enable more objective assessment of adherence. Future analyses should use multilevel or other cluster-adjusted approaches where the number of participating facilities permits reliable estimation of hospital-level effects. Where feasible, future studies should document and compare respondents and nonrespondents and apply appropriate sampling or post-stratification weights when the sample distribution differs from the underlying HCW population. Age and professional experience should also be examined using more detailed, empirically justified categories or as continuous variables where appropriate. Future validation studies should further evaluate the psychometric properties of the questionnaire, particularly the knowledge domains with low internal consistency, and should consider external criteria that would permit ROC-based assessment of outcome thresholds. Finally, studies involving multiple simultaneous hypotheses should prespecify appropriate multiplicity-control procedures, such as the Benjamini–Hochberg false discovery rate approach, to reduce the potential for false-positive findings.

## Conclusion

The findings of this study highlight the need for sustained educational and institutional efforts to improve HCWs’ preparedness for emerging infectious diseases. The identified knowledge gaps emphasize the importance of continuous professional development programs, regular updates on evidence-based guidelines, and effective dissemination of IPC protocols.

Healthcare institutions should implement targeted training strategies tailored to HCWs’ professional roles, educational backgrounds, and levels of clinical experience. Strengthening IPC infrastructure, promoting lifelong learning, and ensuring access to updated clinical resources are essential for enhancing healthcare system resilience.

From a public health perspective, these findings provide useful evidence for policymakers in Yemen and similar resource-limited settings to develop context-specific preparedness plans. Investment in healthcare workforce capacity building and sustainable infection control programs is critical to improve response readiness and reduce the effect of future infectious disease outbreaks.

## Data Availability

The raw data supporting the conclusions of this article will be made available by the authors, without undue reservation.
